# Separator Membrane from Crosslinked Poly(Vinyl Alcohol) and Poly(Methyl Vinyl Ether-alt-Maleic Anhydride)

**DOI:** 10.3390/nano5020398

**Published:** 2015-03-25

**Authors:** Charu Vashisth Rohatgi, Naba K. Dutta, Namita Roy Choudhury

**Affiliations:** Ian Wark Institute, University of South Australia, Mawson Lakes 5095, Australia; E-Mails: charuvashisthrohatgi@gmail.com (C.V.R.); naba.dutta@unisa.edu.au (N.D.)

**Keywords:** poly vinyl alcohol, separator membrane, ionic polymer, interpolymer-networked systems, battery separator, hydrogen bonding, electrochemical impedance spectroscopy, thermal analysis, dynamic mechanical analysis

## Abstract

In this work, we report separator membranes from crosslinking of two polymers, such as poly vinyl alcohol (PVA) with an ionic polymer poly(methyl vinyl ether-alt-maleic anhydride) (PMVE-MA). Such interpolymer-networked systems were extensively used for biomedical and desalination applications but they were not examined for their potential use as membranes or separators for batteries. Therefore, the chemical interactions between these two polymers and the influence of such crosslinking on physicochemical properties of the membrane are systematically investigated through rheology and by critical gel point study. The hydrogen bonding and the chemical interaction between PMVE-MA and PVA resulted in highly cross-linked membranes. Effect of the molecular weight of PVA on the membrane properties was also examined. The developed membranes were extensively characterized by studying their physicochemical properties (water uptake, swelling ratio, and conductivity), thermal and electrochemical properties using differential scanning calorimetry (DSC), dynamic mechanical analysis (DMA), thermo-gravimetric analysis (TGA) and electrochemical impedance spectroscopy (EIS). The DSC study shows the presence of a single *T*_g_ in the membranes indicating compatibility of the two polymers in flexible and transparent films. The membranes show good stability and ion conductivity suitable for separator applications.

## 1. Introduction

A separator is a membrane, whose main function is to keep the positive and negative electrodes apart to prevent electrical short circuits and, at the same time, allow rapid transport of ionic charge carriers by intrinsic ionic conductor [1,2]. It should minimize any processes that adversely affect the electrochemical energy efficiency of the batteries. A variety of separators have been used in batteries over the years. Separators have been manufactured from cellulosic papers and cellophane to nonwoven fabrics, foams, ion exchange membranes, and microporous flat sheet membranes made from polymeric materials. A separator has significant role in all battery systems. The thin membrane performs both as a medium to transport ions during electrochemical reactions and also prevents/isolates the positive and negative electrode from short circuits. They should be chemically and electrochemically inert and should exhibit high mechanical integrity against the electrolyte and electrode materials during cell operation. Current and primary demands on the separator membrane are ion conductivity, excellent mechanical strength, dimensional stability, chemical stability towards oxidation, reduction and hydrolysis, and low cost [2]. For example, to fabricate the separator for a lithium-based battery, the following criteria should be satisfied: (a) electronic insulator; (b) minimal electrolyte resistance (<2 Ω·cm^2^); (c) dimensional stability (shrinkage: <5%); (d) mechanical strength enough to allow the assembly process; (e) chemical stability against degradation by electrolyte or electrode reactants (should be stable for long cycle life); (f) effective prevention of the migration of particles or soluble species between the electrodes; (g) good wettability in electrolyte solution (should be wet completely in liquid electrolytes); (h) uniform thickness and pore distribution (thickness: <25 μm; pore size: <1 μm; porosity: ~40%); increased ionic conductivity, good wettability, and excellent interfacial adhesion between the electrodes and the separators [2]. As batteries have become more sophisticated, the separator function has also become more demanding and complex. Very little work is directed toward developing and characterizing new separators for flow batteries, which can be made readily and scalable. Thus, in this work, we aim to develop a facile route for the fabrication of separators for redox flow batteries, e.g., zinc-bromine.

Amongst a variety of different approaches to synthesize new separator membranes, acid-base polymer blends can offer a potential route to designing improved separator materials due to the interaction between polymers. In multi-component polymer systems, the interfacial interaction between component phases can be effectively utilized to enhance homogeneous processing and achieve unique performance. Such interactions between the component phases can be brought about by either chemical and/or specific interaction between the components or by adding block copolymer of component phases. Amongst various types of specific interactions, hydrogen bonding is an effective route to complexation of two polymers in their blends to result in unique properties. Despite a number of different strategies having been used for membrane development, research on inter polymer ion exchange membranes, prepared from compatible casting solutions of two water soluble polymers is scanty.

A variety of approaches, including thermal cross-linking [3,4,5,6], electrospinning [7,8,9,10,11], chemical cross-linking [12,13,14] are being applied to synthesize new membranes. Although chemical cross-linking is a versatile method to tune polymer microstructure and physical properties; however, to avoid the use of toxic chemicals, environmentally benign routes are gaining importance in membrane fabrication. Among them the acid-base polymer blends have become one of the most facile approach in the design of improved membrane materials due to the interaction (ionically cross-linked) between the polymers.

A series of different polymer blends, such as sulfonated poly(ether ether ketone) (SPEEK) blended with poly(ether imide) (PEI), poly(amide imide) (PAI), and poly(benzimidazole) (PBI), have been reported in the literature [15,16,17] for such a use. In most of the previous studies [3,4], these membranes were used for the desalination of aqueous salt solutions, for various potential in biomedical applications and also for their oxygen barrier properties [18]. It has been observed that the association of a polyacid with polybase can significantly reduce the swelling and the methanol permeability of membranes [19,20] due to stabilization of interpolymer complexes through hydrogen bonding [21]. In addition, unique properties of these complexes include increased impact and tensile strength, which are useful in a wide range of applications.

In this work, poly(vinyl alcohol) (PVA) has been selected as base polymer matrix in view of its film-forming characteristics, hydrophilic properties, and high density of reactive chemical functions that are favorable for cross-linking using thermal treatments [5]. It also exhibits interaction ability with complimentary polymers [22,23]. Several cross-linking methods have been published for different uses. As a rule, all multifunctional compounds capable of reacting with hydroxyl groups can be used to obtain three-dimensional networks in PVA [24]. Poly(methyl vinyl ether-alt-maleic anhydride) (PMVE-MA) was selected due to its very high hydrophilicity and also because it can act as a cross-linking agent for PVA due to the presence of maleic acid groups in its repeating units.

Here we report the preparation of cross-linked membranes, based on PVA and PMVE-MA, and investigate the cross linking process in detail. We also establish the influences of the interaction on the membrane properties including dimensional stability, water uptake, ionic conductivity with increasing PMVE-MA ratios in the blends.

## 2. Experimental Section

### 2.1. Materials

Poly vinyl alcohol (PVA) (98% hydrolyzed, average Mw = 13,000–26,000 g·mol^−1^), (99% hydrolyzed, average Mw = 89,000–98,000 g·mol^−1^) and (99% hydrolyzed, average Mw = 146,000–186,000 g·mol^−1^) and Poly(methyl vinyl ether-alt-maleic anhydride) (PMVE-MA) average Mw = 1,080,000 g·mol^−1^) were procured from Aldrich and were used as received. Hydrochloric acid (98%) from Aldrich was used as esterification catalyst. High-purity Milli-Q water was used throughout the study (resistivity ~18.2 M·Ω·cm).

### 2.2. Blend Membranes

Three different molecular weight PVA samples have been chosen for crosslinking study. The cross-linked PVA/PMVE-MA membranes were prepared using the following two-step procedure. First, aqueous 10 wt% PVA (Table 1) solution was prepared by dissolving a pre-weighed amount polymer at 90 °C for 6 h. Aqueous 10 wt% PMVE-MA solution was prepared by dissolving pre-weighted quantities of dry PMVE-MA in Milli-Q-water at room temperature. Finally, PVA solution was mixed together with PMVE-MA solution at a fixed ratio (Table 1) for 5 h at room temperature until a homogeneous solution was formed. The mixed solution was poured onto a petri dish, and dried at room temperature to prepare the film. The cast polymer solutions were allowed to dry in a vacuum oven at 30 °C for 24 h. The dried membranes were peeled off the Petri dish, and then heated in an oven at 120 °C for 2 h to induce the cross-linking reaction. Insolubilization of the polymer blend films was accomplished via formation of interchain ester crosslinks during heat treatment. The samples were then characterized using spectroscopic, thermal and rheological studies. For all the spectroscopic and thermal studies, we have used blend samples of PVA with a molecular weight 89 K.

**Table 1:** Water uptake and dimensional change of cross-linked PVA/PMVE-MA blend membranes for different molecular weight PVA with various blend ratios. Values are means ± SD.

Sample No. S_PMVE-MA:PVA_	Effect of molecular weight of PVA					
	Mw-13,000–23,000		Mw-89,000–98,000		Mw-124,000–186,000	
	Water uptake (%)	Dimensional change (%)	Water uptake (%)	Dimensional change (%)	Water uptake (%)	Dimensional change (%)
S_10:90_	36	35	42	47	49	50
S_20:80_	19	23	24	25	32	21
S_30:70_	22	26	27	30	39	42
S_40:60_	34	29	37	32	44	41
S_50:50_	40	39	45	42	51	58

## 3. Characterization

### 3.1. Water Uptake and Dimensional Stability

The weight change of the samples was monitored to investigate the water uptake by the membranes. In a typical experiment 10 mm diameter discs were punched out from each sample and the weight of the pre-dried samples was measured (*Wd*_1_). The samples were then immersed in de-ionized water at 25 °C for 24 h, and then surface-attached water on the membranes was removed using a Kim wipe. Then, the wet sample weight (*W_wet_*) was determined as quickly as possible. The weight of dry sample was measured after completely drying the hydrated membranes at 60 °C (*Wd*_2_) for 24 h. The weight change of the membranes was calculated as follow:

(1) Weight change (%) = [(*Wd*_1_ − *Wd*_2_)/*Wd*_1_] × 100

The total water uptake (%) was determined by

(2) Total water uptake (%) = [(*W_wet_* − *W_dry_*)/*W_dry_*] × 100

(3) Dimensional change (%) = [(*D*_wet_ − *D*_dry_)/*D*_dry_] × 100

where *W_wet_*, *D_wet_* and *W_dry_*, *D_dry_* are the wet and dried sample weight and dimensions after immersing in water, respectively. The water uptake measurement was done in triplicate and the average values are reported.

### 3.2. Spectroscopic Analysis

In order to examine the nature of interaction photo-acoustic fourier transform infrared spectroscopy (PA-FTIR) study was performed on a Nicolet Magna-IR Spectrometer 750 (Thermo Fisher Scientific, Waltham, MA, USA) equipped with an MTEC model 300 photoacoustic cell under helium purge with carbon black as the reference. IR spectra were recorded with a wave number resolution of 8 cm^−1^ and 526 scans were performed in the range of 450–4000 cm^−1^. Samples in the form of thin films were placed into the sample holder and placed in the Photo acoustic cell. Carbon black was employed as a background reference.

### 3.3. Cross-Linking of PVA/PMVE-MA by Rheological Measurements

Cross linking kinetics and gel point measurement of PVA/PMVE-MA blend systems were examined using dynamic rheological measurement using a cone-plate geometry (40 mm 2° steel cone) at different temperature range from 30 °C to 45 °C using AR 1000-N Rheometer (TA Instruments, New Castle, DE, USA) and at a single frequency of 1 Hz.

### 3.4. Thermal Analyses

Thermogravimetric analysis (TGA) was done on a TA 2950 Thermal Analyzer (TA Instruments). Samples were dried in an oven at 80 °C prior to analysis and the experiment was performed on approximately 5 mg of the sample under nitrogen with a temperature ramp of 10 °C·min^−1^. Differential scanning calorimetric measurements were done using (DSC) Q2000 (TA Instruments). Each sample was heated from 30 °C to 250 °C at a heating rate of 10 °C·min^−1^ under nitrogen. Then, it was cooled down to 30 °C at the same rate. A second heating was carried out in the same conditions as the first one. The glass transition temperature (*T*_g_) was determined from the second heating scan. The thermal stability of the samples was evaluated in a TA Instrument’s thermogravimetric analyzer at 30–600 °C and 10 °C·min^−1^ under nitrogen/oxygen atmosphere at a flow rate of 50 mL·min^−1^. The degradation temperatures were determined. Dynamic-mechanical analyses (DMA) were performed using a TA Q800 (TA Instruments). It was employed to determine the thermal transitions of the membranes. Scans were carried out at a heating rate of 2 °C·min^−1^ and a frequency of 1 Hz. The loss modulus E' (viscous component) and the storage modulus E'' (elastic component) of the membranes were obtained from the analysis. The *T*_g_s were obtained from the peak of Tan δ (E'/E'') *versus* temperature data.

### 3.5. Conductivity Measurement

The ionic conductivity measurements were carried out at ambient temperature after equilibrating the membrane in de-ionized water for one day. A normal two-point probe technique at relative humidity 95% was used where the membrane sample was sandwiched between the platinum electrodes. Each sample was cut in 4 × 1 cm^2^ prior to mounting on the cell. The impedance spectral measurement of the membrane was carried out using a Solartron Frequency Response Analyzer 1260 A connected to an Electrochemical Interface 1287 (Solartron Metrology, Cambridge, UK). The impedance analyzer worked in galvanostatic mode with AC current amplitude of 0.1 mA over frequency range from 100 kHz to 0.1 Hz. The ionic conductivity (σ) was obtained as follows:

(4) $$\text{σ}=\frac{L}{R\cdot S}$$

where σ is the ionic conductivity (in S/cm); *L* is the distance between the electrodes used to measure the potential (cm); *R* is the impedance of the membrane (in Ω); and *S* is the surface area for ion to penetrate the membrane (in cm^2^).

## 4. Results and Discussion

### 4.1. Interactions Study by PA-FTIR

**Figure 1:**
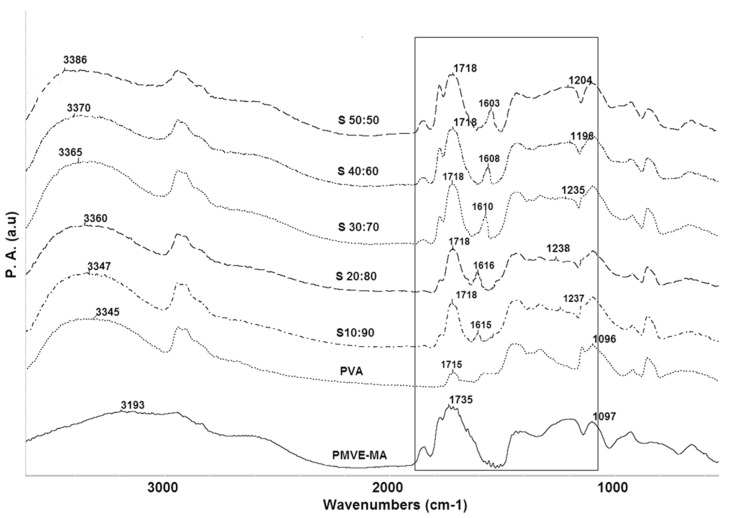
Fourier transform infrared spectroscopy (FTIR) spectra of poly vinyl alcohol (PVA), poly(methyl vinyl ether-alt-maleic anhydride) (PMVE-MA) and cross-linked PMVE-MA/PVA blends. The highlighted area represents the ester bonds formed during cross linking.

FTIR is a powerful tool to investigate specific interaction between polymers. Chemical investigation of the interfacial interaction was performed using PA-FTIR, for all the composition, to gain insight into the degree of inter-polymer complexation that can take place due to hydrogen bonding between carbonyl (C=O) groups of the PMVE-MA and the hydroxyl (–OH) groups of the PVA. Figure 1 represents the FTIR spectra for pristine PVA, PMVE-MA, and PMVE-MA/PVA blend membranes. The spectrum of PMVE-MA shows the presence of a band at 1107 cm^−1^ due to C–O–C group. All the blends show band in the region of 1650–1800 cm^−1^ arising from −C=O group. In pristine PVA, intra-molecular and inter-molecular hydrogen bonds are expected to occur among PVA chains due to high hydrophilic force [25]. The −OH group of pristine PVA (3345 cm^−1^) progressively increases in height after incorporation of PMVE-MA in the blend membranes. This change is caused due to strong interaction between hydroxyl groups on PVA and carboxyl groups on PMVE-MA, and weakening of the hydrogen bonds between PVA chains. Hence, intermolecular interactions are preferred with increasing PMVE-MA ratio, leading to a more effective mixing of both constituents. This observation indicates that the cross linking reaction occurred between PVA and PMVE-MA by creating new covalent bonds and weakening hydrogen bonds between PVA chains. The band in the 1800–1700 cm^−1^ region arises from C=O group vibrations [26]. As seen from Figure 1 (highlighted portion), the bands for −COOH groups show shifting and increase in the intensity after cross-linking when PMVE-MA was initially mixed with PVA, and resulted in the formation of a peak around 1718 cm^−1^ characteristic of COOH group vibration with a gradual increase in the shoulder of the peak which is quite evident from the graph as the PMVE-MA content is increased. The PVA/PMVE-MA blend films show a new absorption band around 1615 cm^−1^ for (C=O) stretching, whereas around 1237 cm^−1^ for (C–O) stretch mode of ester group in sample S**_10:90_**. The absorption peaks for sample S**_30:70_** was around 1610 cm^−1^, 1235 cm^−1^, around 1608 cm^−1^, 1196 cm^−1^ for S**_40:60_** and around 1603 cm^−1^, 1204 cm^−1^ for S**_50:50_**, are assigned as peaks for (C=O) and (C–O) ester group stretching, respectively, and showed shift towards lower wavenumber as we increase the PMVE-MA content [26] due to the formation of ester bonds (C–O–C) between alcohol group of PVA and carboxyl group of PMVE-MA.

### 4.2. Kinetics of Cross-Linking of PVA/PMVE-MA Solutions

**Figure 2:**
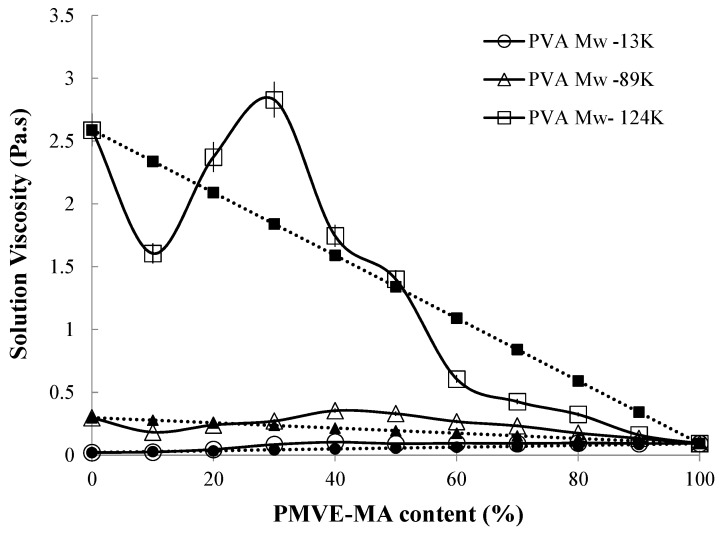
Viscosity of PVA/PMVE-MA blends for different molecular weight PVA as a function of PMVE-MA content by weight, compared to the linear additive viscosity values (dotted lines). Values are means ± SD.

As the strength of interpolymer complexation interactions are strongly determined by the stoichiometric ratios of the component polymers; therefore, optimization of blend ratio is critical. One of the rapid ways of determining the optimum blend ratio and the strength of interactions is through evaluation of blend viscosities. Figure 2 compares the viscosity of each blend with different molecular weight PVA and compares it to the calculated (rule of additivity) value in order to highlight deviations. As observed from the graph, an increase in PMVE-MA content in the blend led to a decrease in blend viscosity due to the dissociation of the carboxylic acid groups. When PVA with Mw-13 K and 89 K was used, a positive deviation in viscosity from linear mixing values for all blend compositions was observed, with a maximum positive deviation of about 0.05 Pa·s and 0.5 Pa·s respectively at 40 wt% PMVE-MA indicating favorable interaction. However, at 10 wt% PMVE-MA resulted in negative deviation from calculated values for PVA Mw-89 K due to decrease in the viscosity of solution. However, further addition of PMVE-MA content leads to positive deviation again. An identical trend was observed with PVA Mw-124 K with the only difference being that the maximum positive deviation was seen ~30 wt% PMVE-MA and, unlike the other two molecular weight PVAs, further addition of PMVE-MA content led to decrease in viscosity until a negative deviation occurred around 60–100 wt% PMVE-MA (positive negative deviation behavior-PNDB). The PNDB phenomenon can be related to concentration-dependent interaction and explained by partial miscibility at low concentration and a concentration-dependent change of the flow mechanism in the resultant blend. The reduction in viscosity and broadening of the peak with a decrease in viscosity can be attributed to higher intramolecular hydrogen-bonding within PMVE-MA, and the inter-polymer interaction is reduced, leading to a reduction in viscosity. The broadening of the peak is probably due to a larger number of possible hydrogen-bonding interactions that can occur at similar energy levels [27].

Various rheological methods have been widely used to determine the gel point of polymer gels. One of the methods involves the use of the peak maximum in tangent angle of mechanical loss (tanδ)_max_. However, the commonly used method is the crossover point to determine the gel point, in part, due to the ease of measurement. Here we have used the later approach for determining the occurrence of the gelation point during crosslinking of PVA/PMVE-MA. The crossover point of G'-t and G''-t curves was studied, *i.e.*, when the system shows the same level of elastic and viscous behavior, meaning that the same quantity of energy is stored and dissipated. In some cases, the intersection point appeared even before the reaction commenced and it is impossible to apply the crossover point of G' and G'', so the peak maximum in tanδ, *i.e.*, where there is maximum difference between the elastic and viscous behavior, is taken as the gel time [28].

**Figure 3:**
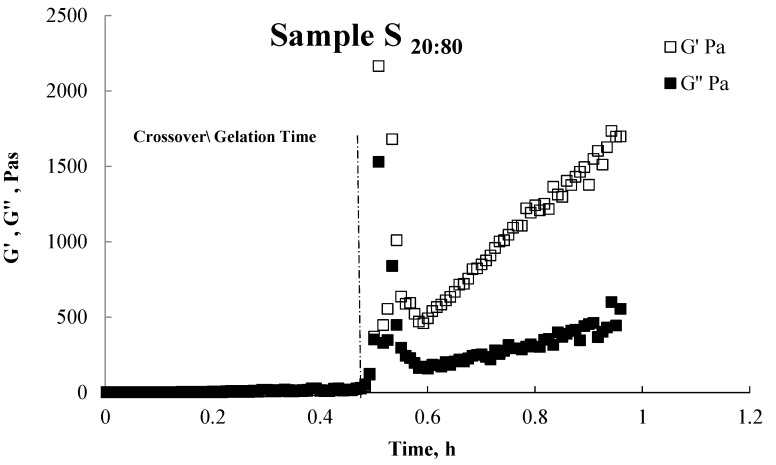
Elastic modulus (G') and viscous modulus (G") of sample S_20:80_ (representative for all ratios) as a function of frequency.

In this system, upon addition of PMVE-MA to PVA solution, the anhydride group first gets hydrolyzed and then reacts with PVA. Figure 3 shows the effect of mixing PVA with PMVE-MA on the elastic modulus (G') and viscous modulus (G'') of a solution blend. For PVA/PMVE-MA solution in sample S**_20:80_** (Figure 3) and at certain temperature the loss modulus G'', is above the elastic modulus G', which demonstrates the relative fluidity of the system, whereas, at the cross over point; G' approaches G'' and almost overlaps. The crossing over point of G' and G'' is the gel time of the sample and after cross over point the G' becomes higher than G'' and is also parallel to G', hence, formation of gel due to the cross linking between PVA and PMVE-MA.

**Figure 4:**
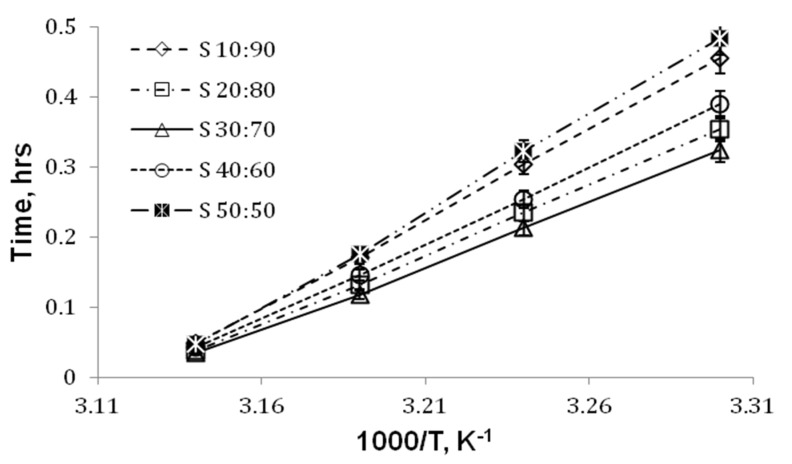
Gelation time *versus* (1000/T), where temperature *T* is expressed in Kelvin. Comparisons of all PMVE-MA/PVA blend solutions. Values are means ± SD.

This trend was observed to be identical for all the samples with different gel times depending on the composition and temperature. Figure 4 represents the effect of temperature and PMVE-MA concentration on the gel time of the blends. Sample S**_40:60_** at room temperature took 0.38 h to gel, whereas it took less than 20 min to gel at a high temperature. This trend was identical with all the blends at high temperature regardless of the PMVE-MA content; however, at room temperature, a difference in gelling time has been observed. The blends at both far ends, *i.e.*, high PVA or high PMVE-MA ratio took more time to form gel as compared to blends with PMVE-MA/PVA ratio in between.

**Scheme 1:**
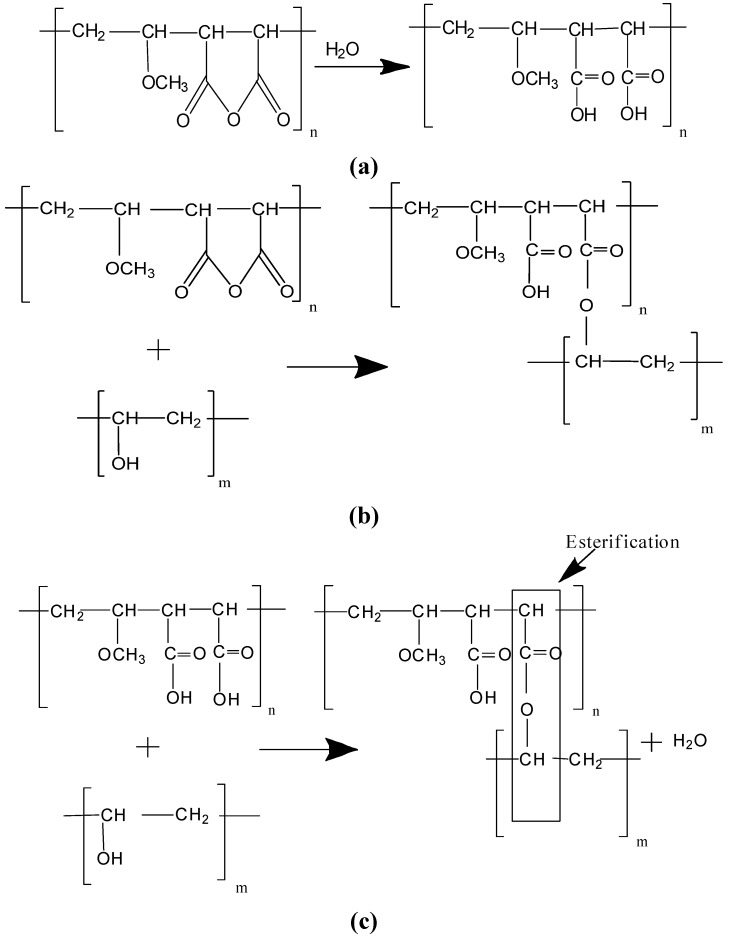
Reaction during mixing of PVA and PMVE-MA (**a**) PMVE-MA with water; (**b**) PVA with partially hydrolyzed PMVE-MA; (**c**) esterification reaction.

The expected cross-linking between the two polymers primarily occurs from the chemical reactions and hydrogen bonding between the hydroxyl groups of PVA with carboxyl groups and ether groups from PMVE-MA. Scheme 1 represents the various proposed reactions occurring during mixing of PVA and PMVE-MA. The anhydride ring of PMVE-MA undergoes hydrolysis in aqueous solution to form free carboxylic acid groups, which further reacts with the PVA. PVA has been reported to crosslink by esterification with di/polyacids, radiation induced chemical reactions. In the present study, the cross-linking reaction has been carried out at a higher temperature for all the samples. The expected cross-linking between the polymers comes from the chemical reactions between hydroxyl groups in PVA with either maleic anhydride groups directly (a) or carboxyl groups (b) from PMVE-MA and also hydrogen bonding between the ether group and hydroxyl groups.

### 4.3. Membrane Characterization by Water Uptake and Dimensional Stability

Table 1 gives the comparison of water uptake and dimensional stability of cross-linked PVA/PMVE-MA blend membranes for different molecular weight PVAs. The presence of water in the membrane facilitates channel for ion transport and increases the mobility of ionic charge carrier. From the table it can be observed that the data reveals a parabolic trend, *i.e.*, the minimum is indicated by the blend ratio S**_30:70_** for all molecular weight PVAs. It can be explained by the fact that incorporation of 20%–40% PMVE-MA in the blend is leading to optimum water uptake, whereas below and above this ratio it causes excessive swelling, indicating that 60%–80% PVA is needed in order to attain maximum interaction between PVA and PMVE-MA, which leads to controlled water uptake and dimensional stability. Then, at low concentrations of PMVE-MA; PVA intermolecular interactions are preferred and only when the PVA concentration is sufficiently high does the intermolecular interaction becomes prominent [29]. As can be observed from Table 1, the water uptake behavior of the films was affected by the ratio of PVA to PMVE-MA content in the blends. When the ratio of PMVE-MA is high compared to PVA it leads to highly water swollen membranes. For each molecular weight a similar trend was observed, which reveals better interaction of low molecular weight PVA compared to high molecular weight PVA. This can be due to PMVE-MA moiety being much larger than the PVA and introduction of the PMVE-MA moieties increases the interchain space within the PVA matrix.

Therefore, more free volume is available for the sorbed water molecules. In addition, dimensionally stable membranes resulted at optimum ratios of PVA/PMVE-MA in the blends regardless of the PVA molecular weight as more −COOH groups are available for the cross linking with −OH groups of PVA and increased hydrogen bonding results in reducing the mobility of the polymer chains, hence better stability also evident from the FTIR data. However, it was also observed that if a higher ratio of PMVE-MA was used than that of PVA; it increases the brittleness of the membrane, even in the water swollen membranes [4]. From Table 1 it is clear that PVA with Mw-13 K has most efficiently cross-linked with PMVE-MA followed by PVA Mw-89 K and PVA Mw-124 K, *i.e.*, the smaller the molecular weight of PVA the more stable it is. This observation can be explained in that with smaller molecular volume, a relatively easily accessible hydroxyl groups per unit mass favoring the complexation. Therefore, the low molecular-weight PVA may have diffused into, and interacted more effectively with, the PMVE-MA chains resulting in restricting their mobility and reducing the swelling of the polymer matrix. Furthermore, it is clear that lower molecular weight PVA has increased miscibility with the PMVE-MA polymer molecules, which may have resulted in a further increase in cross-linking efficiency as observed with other polyalcohol [30,31].

### 4.4. Thermal Properties

**Figure 5:**
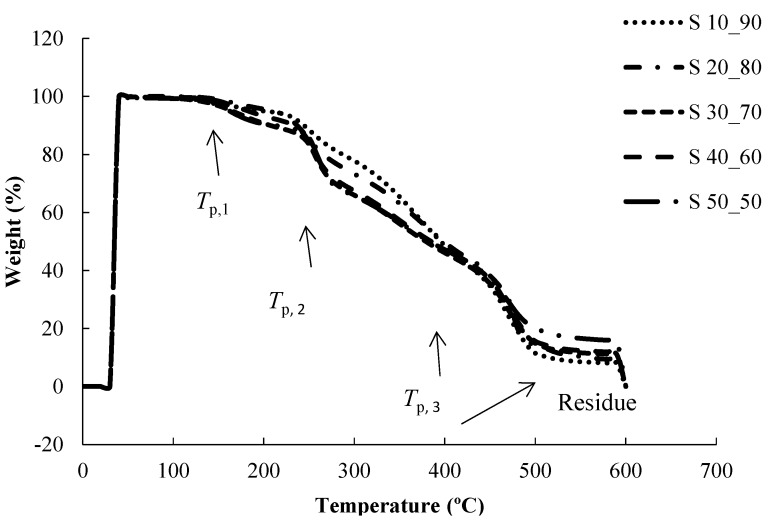
TGA thermograms of the PVA/PMVE-MA interpolymer membranes.

Figure 5 shows the TGA thermograms of the PVA/PMVE-MA interpolymer membranes with various PMVE-MA ratios. TGA curves of the PVA/PMVE-MA polymer films revealed three main weight loss regions. The first region, at a temperature of 100–200 °C (*T*_p,1_ at 124 °C), degradation of PVA polymer chains; the weight loss of the membrane was about 2.0–3.0 wt%. The second transitional region, at around 210–300 °C (*T*_p,2_ at 240 °C), appeared to be due to the degradation of the side-chain of the PMVE-MA polymer membrane; the total weight loss corresponding to this stage was about 11–12 wt%. The peak of the third stage at 295 °C (*T*_p,3_ at 295 °C) occurred due to the cleavage of ester groups of PVA/PMVE-MA composite polymer membrane with a total weight loss at about 90 wt% at 600 °C. The degradation peaks of the cross-linked PVA/PMVE-MA composite polymer samples were less intense and shifted towards higher temperatures. The weight loss results of the samples are recorded at various temperatures in Table 2. It could, therefore, be concluded that the thermal stability was improved due to addition of PMVE-MA and the chemical crosslinking reaction between the −OH groups on the PVA and the −COOH group on the PMVE-MA.

**Figure 6:**
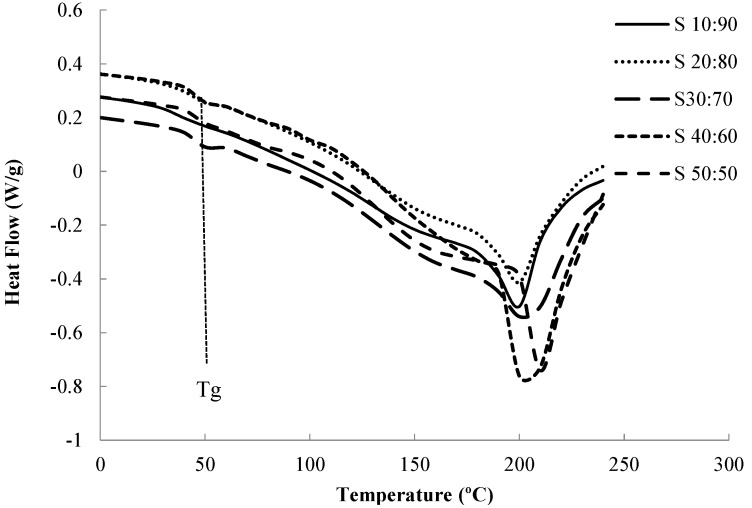
DSC thermograms of PMVE-MA/PVA blends.

PVA, PMVE-MA, and PVA/PMVE-MA samples were also analyzed by differential scanning calorimetry. The DSC measurements were carried out by a heating-cooling-heating cycle. The purpose of the first heating cycle was to remove any thermal history of the PVA composite polymer membrane. The DSC curves for the pure PVA polymer and the PVA/PMVE-MA membranes with various PMVE-MA compositions are shown in Figure 6.

**Table 2:** TGA results of weight loss of the PVA/PMVE-MA blend polymer membranes at various temperatures.

S_PMVE-MA:PVA_ Blends	Weight Loss (%) at different temperature				
	200 °C	300 °C	400 °C	500 °C	600 °C
S_10:90_	4.31	16.57	52.70	88.93	92.10
S_20:80_	5.23	21.98	51.76	86.65	90.23
S_30:70_	6.18	25.29	52.28	84.26	88.24
S_40:60_	7.64	30.07	49.38	78.60	84.26
S_50:50_	9.92	30.69	55.81	85.51	88.83

The *T*_g_ of a polymer represents the amorphous regions of a polymer and is, therefore, useful to reflect changes that occur in these regions. From Table 3, it was observed that only one *T*_g_ is found in the dry samples, indicating miscibility over entire composition. It had been reported that the *T*_m_ and *T*_g_ of the pure PVA polymer with 98%–99% hydrolysis degree are at 226 °C and 81.59 °C, respectively [32]. It was found that the glass transition temperature (*T*_g_) and the melting temperature *T*_m_, decreased from 81.59 to 40.46 °C, and from 226 °C to 199 °C, respectively, in sample S_10:90_, and increased again with increasing PMVE-MA content in the samples. A change of *T*_g_ and *T*_m_ of PVA/PMVE-MA composite polymer membrane indicated a change between a semi-crystalline phase and an amorphous phase and the decrease in *T*_g_ and *T*_m_ is due to plasticization effect at this lower ratio (S_10:90_).

**Table 3:** DSC results of PVA and PMVE-MA blends.

Temperature (°C)	PVA	PMVE-MA	Blend membranes				
			S_10:90_	S_20:80_	S_30:70_	S_40:60_	S_50:50_
***T*_g_**	81	77	40	42	44	47	49
***T*_m_**	226	163	199	199	204	204	209
**∆H**	57	406	154	141	189	164	216

### 4.5. Conductivity

**Figure 7:**
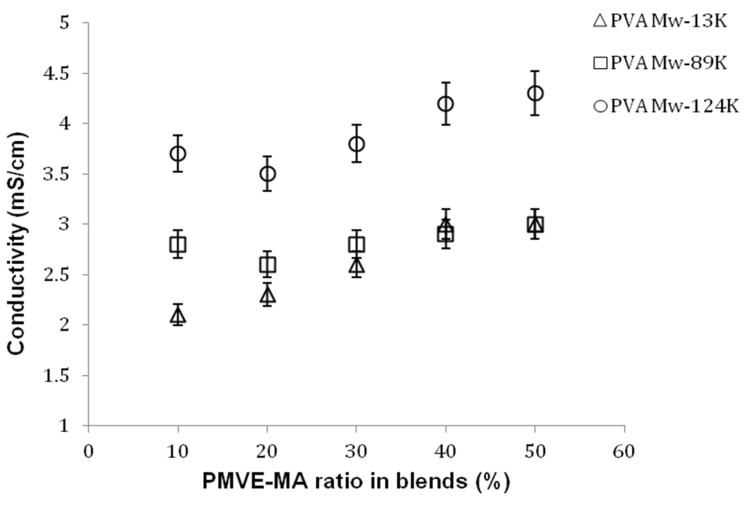
Effect of PMVE-MA ratio and PVA molecular weight on the conductivity of the blend membranes. Values are means ± SD.

To use a polymer membrane separator it is necessary to provide a facilitated channel for ion transport by providing ionic exchange sites, such as carboxylic acid. During the thermal treatment the hydrogen bonds between –OH of PVA and –COOH of PMVE-MA are formed due to the proximity of the polymer chains [15]. This physical interaction between the functional groups results in the formation of hydrophilic ionic channels or micro-domains by the arrangement of hydrophilic polymeric groups. Figure 7 represents the effect on the conductivity of the membrane as the PMVE-MA ratio is increased. Since the conductivity under the conditions used (room temperature and 100% humidity) are largely ascribed to the movement of free water within the material, a systematic trend in conductivity is expected with blend interactions and water uptake behavior. When the ratio of PMVE-MA in the system is increased, a gradual increase in the conductivity of the membrane was observed. This trend is identical with different molecular weights of PVA used. This increase is due to the presence of several ion acceptor site contained in each PMVE-MA unit compared to the one ion donor of the PVA repeat unit. As the ionic conductivity is dependent on the state of hydration of the membrane, as in a water-swollen membrane, the ions are solvated and enabled to transport through the membrane, which can be easily seen from Figure 7. The crosslinked samples with high molecular weight PVA show two times higher (~4.5 mS/cm) conductivity values as compared to highly cross-linked blends with low molecular weight PVA due to excellent strength of interaction and reasonable amount of water sorption.

## 5. Conclusions

This study confirmss that both PVA and PMVE-MA are able to form excellent membranes from homogenous and miscible solutions as a result of inter-polymer complexation that takes place between the carboxylic acid groups of PMVE-MA and the hydroxyl groups of PVA. The carboxylic acid (*i.e.*, hydrolyzed PMVE-MA) in this work served two purposes: to provide effective cross-linking and, at the same time, to impart a fixed charge character to the cross-linked membranes. The charge character results from the presence of carboxylate groups of the hydrolyzed but unesterified PMVE-MA. This was first evidenced by insolubility and dimensional stability of the blend films in water. FTIR results confirm the presence of these hydrogen bonding interactions by the appearance of two new bands at ~1600 and ~1200 cm^−1^ characteristic of the ester groups arising from the C=O and C–O groups, respectively. The appearance of a single *T*_g_ for each of the blends by DSC proves that miscible blends were formed, resulting from the hydrogen bonding. The chemical interaction between the functional groups results in the formation of hydrophilic ionic channels or micro domains by the arrangement of hydrophilic polymeric groups leading to an increase in ionic conductivity in membranes. The optimized membranes from PVA and PMVE-MA show good ion conductivity making them suitable for separator applications.
